# The Effects of Multivitamin Supplementation on Diurnal Cortisol Secretion and Perceived Stress

**DOI:** 10.3390/nu5114429

**Published:** 2013-11-11

**Authors:** David A. Camfield, Mark A. Wetherell, Andrew B. Scholey, Katherine H. M. Cox, Erin Fogg, David J. White, Jerome Sarris, Marni Kras, Con Stough, Avni Sali, Andrew Pipingas

**Affiliations:** 1 Centre for Human Psychopharmacology, Swinburne University, Melbourne 3122, Australia; E-Mails: david.camfield@gmail.com (D.A.C.); ascholey@swin.edu.au (A.B.S.); kcox@swin.edu.au (K.H.M.C.); Erin.fogg@gmail.com (E.F.); dawhite@swin.edu.au (D.J.W.); jsarris@unimelb.edu.au (J.S.); marni.kras@monash.edu (M.K.); cstough@gmail.com (C.S.); 2 Stress Research Group, Department of Psychology, Northumbria University, Newcastle Upon Tyne NE1 8ST, UK; E-Mail: mark.wetherell@northumbria.ac.uk; 3 Department of Psychiatry, The University of Melbourne, Melbourne 3000, Australia; 4 National Institute of Integrative Medicine, Melbourne 3123, Australia; E-Mail: asali@niim.com.au

**Keywords:** cortisol awakening response, perceived stress, multivitamins, B vitamins, homocysteine

## Abstract

Recent evidence suggests that dietary intake of vitamins, in particular the B-vitamins including B6, B9 and B12 may have a number of positive effects on mood and stress. Given the effects of stress on a range of biological mechanisms including the endocrine system, it could be reasonably expected that multivitamin supplementation may also affect markers of these mechanisms such as diurnal cortisol secretion. In the current double-blind placebo-controlled study 138 adults (aged 20 to 50 years) were administered a multivitamin containing B-vitamins *versus* placebo over a 16-week period. Salivary cortisol measurements were taken at waking, 15-min, 30-min and at bedtime, at baseline, 8-weeks and 16-weeks. Perceived Stress (PSS) was measured at baseline, 8-weeks and 16-weeks, while blood serum measures of B6, B12 and homocysteine (HCy) as well as red cell folate (B9) were also collected at these time points. A significant interaction was found between treatment group and study visit for the Cortisol Awakening Response (CAR). Compared to placebo, at 16-weeks multivitamin supplementation was found to be associated with a near-significant trend towards an increased CAR. No significant differences in PSS were found between groups, with PSS increasing in both groups across the course of the study. Red cell folate was found to be significantly correlated with the CAR response at 16-weeks while HCy levels were not found to be associated with the CAR response, although HCy significantly correlated with waking cortisol levels at 8-weeks. A possible interpretation of the elevation in CAR associated with multivitamin supplementation is that this represents an adaptive response to everyday demands in healthy participants.

## 1. Introduction

The hypothalamic-pituitary-adrenal (HPA) axis is the primary mechanism by which the hormonal system interacts with the central nervous system. In response to environmental stressors the HPA axis mobilizes metabolic resources to meet increased energetic demands [1]. The final effector hormone of the HPA axis is cortisol, with the HPA axis controlling cortisol levels in a diurnal pattern of secretion. Typically cortisol release increases markedly upon wakening (Cortisol Awakening Response: CAR), up to 50%–70% within the first 30 min, and then gradually declines throughout the day, reaching a nadir around midnight [2,3]. Deviations from this diurnal pattern of cortisol secretion have been found to be associated with a number of negative consequences for health, including sleep disorders [4] and chronic illness [5]. This raises the possibility that alterations in patterns of diurnal cortisol section may be a mediating factor which links subjective reports of chronic stress with physical health problems [6].

The effect of specific dietary nutrients on diurnal cortisol secretion patterns is an area of research that is yet to be adequately addressed; yet preliminary investigations suggest that diet and nutritional status may be an important factor. Garcia-Prieto *et al*. [7] in a study of 41 women from the Mediterranean region, reported that women with a higher dietary intake of monounsaturated fatty acids and a lower intake of saturated fatty acids displayed a greater variability in diurnal cortisol secretion, characterized by a steeper decline in cortisol secretion from morning to night. Similarly, Heaney *et al*. [8] measured salivary cortisol over the course of one day in young students (aged 18–22 years) in comparison to older adults (aged 65–88 years) and found that in general the older adults displayed significantly reduced cortisol upon awakening, a lower cortisol awakening response, and a flatter diurnal profile across the course of the day. However, importantly, Heaney *et al*. [8] also found that for the young students who consumed a diet consisting of *high* fat and *lower* fruit and vegetable intake, a flatter diurnal profile was displayed that resembled the older adults [8]. In contrast, a more recent study by Michels *et al*. [9] in children aged 5–10 years old provided evidence to suggest that frequent consumption of sweet foods, rather than fatty food, was associated with a larger CAR in the mornings.

There is reason to believe that the dietary intake of vitamins, in particular the B-vitamins including B6 (pyridoxine), B12 (cobalamin/cyanocobalamin) and B9 (folate) may also influence patterns of diurnal cortisol secretion. In a study whereby cortisol was experimentally administered to humans over a four day period, resultant decreases in serum concentrations of folate as well as cobalamines were observed [10]. While little is known about causation in the opposite direction, evidence suggests that multivitamin supplementation containing B-vitamins may have a number of positive effects on mood, cognition and general health [11,12,13,14]. In particular, recent studies have reported that supplementation with high dose B vitamins may have efficacy in ameliorating symptoms of stress. For instance, in a sample of 300 otherwise-healthy adults Schlebusch *et al*. [15] reported that ratings of subjective stress were significantly improved following four weeks supplementation with a vitamin B complex. Similar findings were reported in a smaller study of 80 healthy males by Carroll *et al*. [16], whereby ratings on the perceived stress scale (PSS) were significantly reduced following four weeks of supplementation with Berocca^®^. More recently, Haskell *et al*. [17] reported that nine weeks supplementation with a multivitamin in younger women led to reductions in fatigue and improvements on the multi-tasking stressor test [18], while in healthy older men, Harris *et al*. [19] reported that eight weeks supplementation with a multivitamin brought about significant reductions in scores on the Depression Anxiety Stress Scale. In a workplace setting there is also evidence of efficacy for B vitamins in alleviating chronic stress, with a recent study by Stough *et al*. [20] reporting that three months supplementation with a high dose vitamin B-complex resulted in significantly lower ratings of personal strain on the Occupational Stress Inventory (OSI-R). Rucklidge *et al*. [21] reported that multinutrient supplementation may also improve emotional recovery following a substantial acute stressor. Earthquake survivors who received four weeks of multivitamin supplementation showed significantly greater reductions in distress related to the event and the prevalence of probable PTSD, than those who received no treatment. Supplementation was also associated with significantly greater improvements in mood, anxiety and stress. Finally a recent meta-analysis by Long and Benton [22] concluded that supplementation with multivitamin/mineral, particularly those with high dose B vitamins, has a beneficial effect on perceived stress, mood and mild psychiatric symptoms in healthy individuals.

One mechanism by which multivitamin/B vitamin supplementation may help in reducing ratings of stress and fatigue is through the clearance of homocysteine (HCy). HCy is produced in the human body as a result of methionine metabolism, a process which is important for the methylation of a wide range of substances, including DNA [23]. The B vitamins, in particular folate, B6 and B12, are necessary in order to convert HCy back to methionine. If HCy is not metabolized back to methionine in sufficient quantities methylation will be inhibited. Further, accumulation of HCy has been found to be a cause of oxidative stress, DNA strand breakage and mitochondrial membrane damage [23,24]. The link between HCy and stress has also been well documented, with Kang *et al*. [25] reporting a significant relationship between chronic job-related stress and plasma HCy levels and Stoney *et al*. [26] reporting a significant relationship between acute psychological stress and increased HCy in plasma. In addition to the role that B-vitamins play in reducing HCy levels, both folate and B-12 also have direct effects on mood and neurotransmitter regulation through the synthesis of *S*-adenosyl-methionine (SAMe) [27]. The role of SAMe in the pathophysiology of depression as well as its efficacy as an anti-depressant have now been well documented, with recent reviews concluding that SAMe taken orally for 4-weeks or more has equivalent efficacy to tricyclic antidepressants [28,29].

In the current study, the relationship between diurnal cortisol secretion, stress and chronic multivitamin supplementation was examined for the first time in healthy adults using a double-blind placebo-controlled design. The primary outcome measures under investigation were the changes in diurnal cortisol secretion patterns at 16-weeks in response to multivitamin supplementation. As additional exploratory analysis, correlations between salivary cortisol parameters and perceived stress, blood measures of B vitamins and HCy were also examined at both baseline, 8-weeks and 16-weeks in order to further explore relationships between nutritional status, stress and HPA function.

## 2. Methods

### 2.1. Participants

A total of 138 participants (78 Females and 60 Males) aged between 20 and 50 years (M = 30.79, SD = 6.99) were recruited via advertisements in newspapers and flyers, as well as radio, television and social media. The current study formed part of a larger investigation regarding the effects of multivitamin supplementation on cognition and mood, with these results published elsewhere [30]. The sample size that was used for the saliva cortisol analysis was the same sample size that was used for the larger study, and was determined on the basis of power analysis conducted using previous research regarding the effects of multivitamin supplementation on general health [19]. Due to the lack of previous research regarding the effect of chronic multivitamin supplementation on diurnal cortisol patterns, the current study was considered to be exploratory. However, using G^*^Power 3.1 the sample size was found to be sufficient for detecting significant interactions between time and treatment groups of small effect size (*f* = 0.1) with 80% probability, assuming an alpha level of *p* = 0.05. The study was also found to be powered adequately to detect significant interactions within each male and female subgroup of small-medium effect sizes (*f* = 0.150 and *f* = 0.165 respectively) with 80% probability using an alpha level of *p* = 0.05.

**Figure 1:**
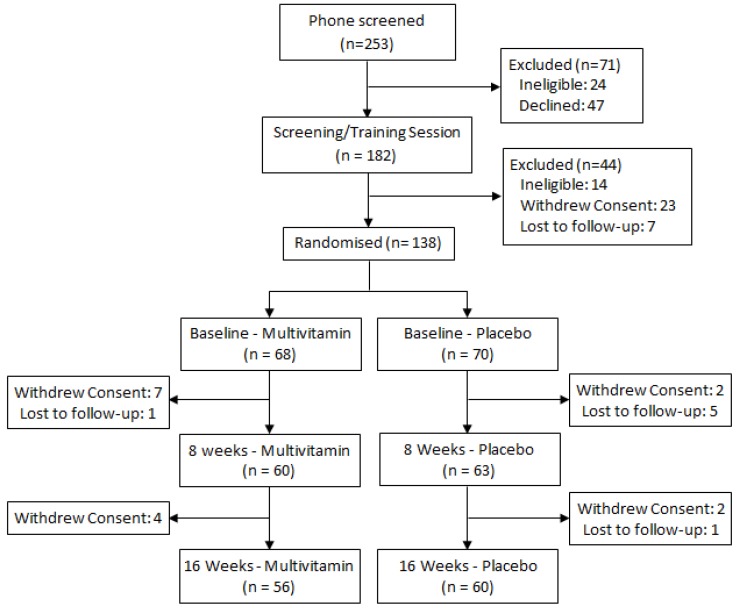
Trial profile.

Participants were required to be engaged in at least part-time employment or study and to have no history of head injury, stroke, psychiatric or neurological conditions, heart disease, diabetes or any other condition that would impair food metabolism. The Beck Depression Inventory II (BDI-II) was additionally completed by all participants at enrolment in order to screen for symptoms of clinical depression, with a cut-off score of 20 used as an exclusion criteria. Female participants were also excluded if they were pregnant, and no participant was permitted to be taking any other herbal or nutritional supplement or prescription medication, with the exception of the contraceptive pill, for the duration of the study. All participants provided written informed consent and the study was approved by the Swinburne University Ethics Committee and listed with the Australia-New Zealand Clinical Trials Registry (SUHREC Project 2010/261, ANZCTR #12611000092998). The CONSORT trial profile is displayed in Figure 1.

### 2.2. Treatments and Randomization

The study was a 16-week, double-blind placebo-controlled parallel groups design. Participants were randomized to receive either Swisse Ultivite Formula 1^®^ (Men’s/Women’s formula) multivitamin or placebo. Treatment randomization was conducted separately for Males and Females by the Supplier: Swisse Vitamins Pty Ltd. Participants were provided with enough treatment for the duration of the 16-week trial, and were instructed to take one tablet daily with, or immediately following breakfast. The Swisse multivitamin major ingredients include B vitamins as well as vitamins C, D and E, together with select mineral chelates and small quantities of select botanicals. Quantities of B vitamins were as follows: B6 pyridoxine hydrochloride 30 mg (men’s)/50 mg (women’s), B9 folic acid 500 μg (men’s)/500 μg (women’s), B12 cyanocobalamin 30 μg (men’s)/50 μg (women’s). Both multivitamin preparations contained a blend of vitamins exceeding recommended daily intakes (RDI) for Australia [31], as well as minerals such as calcium, magnesium, potassium and iron. The multivitamin preparations also contain a range of antioxidants and medicinal herbs including *Ginkgo biloba*, *Panax*/*Siberian Ginseng*, *Vitis vinifera*, *Silbybum marianum* (St. Mary’s thistle) and *Camellia sinensis* (Green Tea). Though the two formulations are roughly equivalent, the amounts of certain nutrients vary slightly, e.g., the women’s formula contains higher levels of calcium and iron, and there are a small number of herbal plant extracts unique to each preparation. A complete list of the Men’s and Women’s Ultivite Formula 1^®^ ingredients can be obtained from the corresponding author. The placebo tablets were matched in size and color to the multivitamin tablets, and included a small amount of riboflavin (2 mg) so as to provide a similar urine coloration effect.

### 2.3. Salivary Cortisol Collection

Participants were required to attend study visits at three separate times: Baseline (week-1), week-8 and week-16. On the day before each scheduled study visit, participants were required to collect four saliva samples using Salivette^®^ collection kits; waking, 15 min after waking, 30 min after waking and then just before going to bed that night. Participants were asked to chew sterile cotton swabs for 1–2 min before depositing the swabs into collection tubes, once saturated. They were also instructed to refrigerate the collected samples until they were returned on the following day and record the times at which each of the samples were taken. In order to monitor adherence to the saliva sampling protocol, participants were instructed to record waking and sampling times as accurately as possible. In order to maintain the integrity of CAR samples any of the 15 min samples that were collected more than 30 min after waking, and any 30 min samples that were collected more than 60 min after waking were excluded from analysis. On attending each subsequent study visit saliva samples from the previous day were returned to the lab, where they were immediately stored at −20 °C. At the conclusion of the 16-week study all samples were thawed and analyzed using high sensitivity EIA Salivary Cortisol EIA kits (Salimetrics), with intra- and inter-CVs being less than 10%.

### 2.4. Blood Collection

Blood collection was collected at baseline as well as at 8-weeks and at 16-weeks. Participants were required to fast from 10 pm the previous night, and were scheduled to arrive at the Centre for Human Psychopharmacology between 8:30 am and 9:30 am for a fasting blood sample on the following morning. A blood sample was collected via venipuncture by a nurse or trained researcher. A total of 43 mL of blood was taken which included the following: 8.5 mL collected in serum separator tube containing clot activator (silicone and micronized silica), for analysis of HCy and vitamin B12 levels (among other tests), the blood was left to clot at room temperature before being centrifuged; 4 mL collected in tube containing anticoagulant (heparin) for analysis of vitamin B6 levels, this tube was wrapped in foil to prevent degradation of the sample by light and; 4 mL in a tube containing anticoagulant (ethylenediaminetetraacetic acid, EDTA) for analysis of red cell folate levels. Samples were sent by courier to a commercial pathology lab for analysis.

### 2.5. Perceived Stress

The Perceived Stress Scale (PSS) [32] is a 10-item questionnaire which measures perceived levels of stress over the previous month. Scale responses range from 0, never to 4, very often, with higher scores indicating greater levels of perceived stress. Participants completed the PSS scale at baseline, 8-weeks and 16-weeks while attending the laboratory, the day following their saliva cortisol sampling. The PSS is a chronic, trait measure of psychological distress and for this reason was expected to remain relatively stable from day to day [33]. Reliability was found to be acceptable across all study visits. Baseline: α = 0.860, 8-weeks: α = 0.867, 16-weeks: α = 0.715.

### 2.6. Statistical Analysis

One-way analysis of variance (ANOVA) revealed no significant differences between the placebo and multivitamin groups on age or body mass index, therefore these variables were not included in subsequent analysis regarding saliva cortisol parameters. Salivary cortisol values in nmol/L were transformed prior to analysis using box-cox power transformations in cases where the data was found to be significantly skewed. Repeated measures ANOVA, considering treatment group (multivitamin *versus* placebo) as the between-subjects variable and time of day (waking, 15 min, 30 min and bedtime) as the within-subjects variable was conducted for each study visit. Separate repeated measures ANOVA were also conducted for waking and evening salivary cortisol levels using treatment group (multivitamin *versus* placebo) as the between-subject variable and Study Visit (baseline, 8-weeks, 16-weeks) as the within-subjects variable. The Cortisol Awakening Response (CAR) was calculated for each participant at each study visit using the maximum value out of the 15 min and 30 min sample minus the waking value for that day. Repeated measures ANOVAs were conducted for CAR using treatment group (multivitamin *versus* placebo) as the between-subject variable and study visit (baseline, 8-weeks, 16-weeks) as the within-subjects variable. Repeated measures ANOVA was also used to assess changes in PSS scores using treatment group (multivitamin *versus* placebo) as the between-subject variable and study visit (baseline, 8-weeks, 16-weeks) as the within-subjects variable. All analyses were conducted for the sample as a whole as well as for males and females separately. All analysis of variance was conducted using mixed linear modeling (PROC MIXED) in SAS version 9.2 [34], with an unstructured variance-covariance matrix fitted to the within-subjects (repeated) variable. Due to the exploratory nature of the current study, no adjustments were made for multiple comparisons, with *p*-values < 0.05 associated with main effects or interactions discussed as significant findings in text. Differences of Least Square Means were subsequently reported in cases of significant main effects and interactions in order to test for group differences associated with specific time points. Differences of Least Square Means with *p*-values > 0.05 and <0.10 are discussed in text as trend-level. Cohen’s *d* effect sizes were calculated for significant mean differences (*p* < 0.05) on the basis of *t*-test values obtained from differences of Least Square Means. Separate formulas were used for (A) repeated (dependent) measurements taken from the same treatment group, *versus* (B) measurements taken from independent groups: [35]

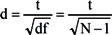
(A)

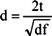
(B)
*t* = *t*-Test value for difference of least square means, *df* = *t*-test degrees of freedom, *N* = number of paired comparisons.

In order to investigate the baseline relationship between perceived stress and saliva cortisol secretion, participants were divided into high and low stress groupings on the basis of their PSS score (Low: 1–14, High: 16–33) [6,33]. Independent *t*-tests were used to test for significant differences on salivary cortisol parameters between high and low PSS groups at baseline. Further exploratory analyses were also conducted in the sample as a whole at baseline, 8-week and 16-week time-points, whereby zero order and partial correlations were calculated between the various B-vitamins, HCy, PSS and salivary cortisol parameters (waking cortisol, evening cortisol and CAR). An alpha level of *p* = 0.05 was also applied to these analyses.

## 3. Results

### 3.1. Primary Analysis: Changes in Salivary Cortisol Parameters in Response to Multivitamin Supplementation

**Figure 2:**
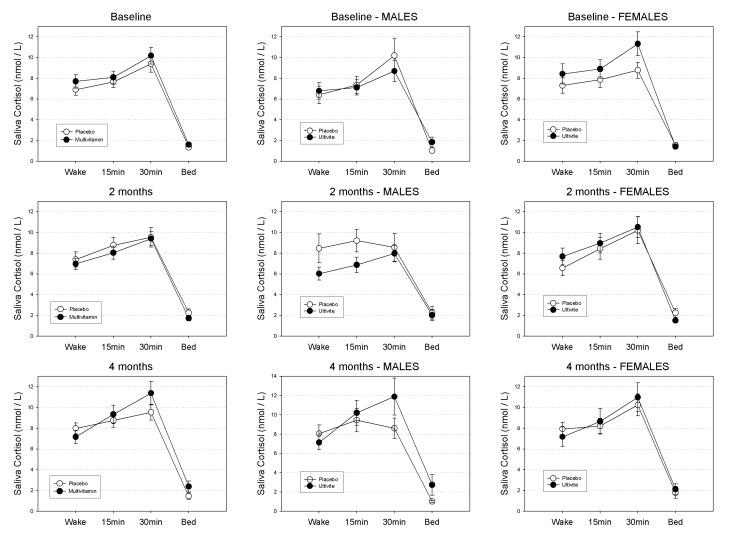
Mean Diurnal Cortisol Secretion (nmol/L) quantities in the total sample at baseline, 8-weeks and 16-weeks for Placebo and Multivitamin groups. Standard Errors of the Means (SEM) displayed as error bars.

Salivary cortisol untransformed means (±SEs) in nmol/L at waking, 15 min, 30 min and evening across the course of the study are displayed in Table 1 according to gender and treatment group. Patterns of diurnal cortisol secretion according to treatment group in the sample as a whole are graphically displayed in Figure 2.

**Table 1:** Salivary cortisol in nanomoles per liter (M ± SE) at waking, 15 min, 30 min and bedtime for baseline, 8-week and 16-week study visits across Placebo and Multivitamin groups*.*

Study Visit/Assay Time			Total		Males		Females	
			*N*	M ± SE	*N*	M ± SE	*N*	M ± SE
**Placebo**								
	*baseline*	Wake	67	6.908 ± 0.55	29	6.396 ± 0.836	38	7.299 ± 0.74
		15 min	66	7.653 ± 0.56	28	7.353 ± 0.846	38	7.873 ± 0.76
		30 min	65	9.388 ±0.816	28	10.20 ± 1.604	37	8.772 ± 0.77
		Bedtime	58	1.337 ± 0.15	23	1.020 ± 0.149	35	1.546 ± 0.22
	*8-weeks*	Wake	60	7.383 ± 0.74	26	8.460 ± 1.399	34	6.559 ± 0.74
		15 min	60	8.763 ± 0.75	25	9.207 ± 1.082	35	8.446 ± 1.04
		30 min	57	9.534 ± 0.94	23	8.556 ± 1.342	34	10.20 ± 1.30
		Bedtime	48	2.248 ± 0.35	17	2.257 ± 0.615	31	2.244 ± 0.44
	*16-weeks*	Wake	53	7.983 ± 0.52	23	8.052 ± 0.897	30	7.930 ± 0.63
		15 min	53	8.749 ± 0.66	23	9.444 ± 1.196	30	8.216 ± 0.73
		30 min	53	9.540 ± 0.75	23	8.616 ± 1.046	30	10.25 ± 1.06
		Bedtime	49	1.453 ± 0.31	21	1.025 ± 0.105	28	1.774 ± 0.53
**Multivitamin**								
	*baseline*	Wake	61	7.696 ± 0.66	27	6.788 ± 0.789	34	8.418 ± 0.99
		15 min	62	8.093 ± 0.60	28	7.117 ±0.735	34	8.897 ± 0.89
		30 min	60	10.18 ± 0.80	26	8.694 ± 1.024	34	11.32 ± 1.16
		Bedtime	56	1.597 ± 0.23	24	1.840 ± 0.473	32	1.415 ± 0.20
	*8-weeks*	Wake	53	6.962 ± 0.54	23	6.024 ± 0.617	30	7.682 ± 0.81
		15 min	55	8.049 ± 0.64	24	6.865 ± 0.743	31	8.966 ± 0.95
		30 min	55	9.399 ± 0.69	24	7.970 ± 0.795	31	10.51 ± 1.02
		Bedtime	53	1.739 ± 0.27	24	2.024 ± 0.546	29	1.504 ± 0.21
	*16-weeks*	Wake	53	7.160 ± 0.62	23	7.140 ± 0.767	30	7.176 ± 0.93
		15 min	51	9.335 ± 0.89	22	10.22 ± 1.295	29	8.661 ± 1.23
		30 min	54	11.38 ± 1.14	24	11.89 ± 1.913	30	10.98 ± 1.39
		Bedtime	47	2.384 ± 0.53	20	2.732 ± 1.067	27	2.126 ± 0.51

For the sample as a whole: at baseline neither the main effect for treatment (*F*(1,131) = 1.15, *p*-value = 0.29), or the treatment by time of day interaction were found to be significant (*F*(3,131) = 1.15, *p*-value = 0.29). Similarly, at 8-weeks neither the treatment effect (*F*(1,115) = 0.31, *p*-value = 0.58) or the treatment by time of day interaction were significant (*F*(3,115) = 0.69, *p*-value = 0.56). At 16-weeks the treatment effect was non-significant (*F*(1,106) = 0.32, *p*-value = 0.58) while the treatment by time interaction was approaching significance (*F*(3,106) = 2.14, *p*-value = 0.099).

For Males: at baseline neither the main effect for treatment (*F*(1,57) = 0.27, *p*-value = 0.60), or the treatment by time of day interaction were found to be significant (*F*(3,57) = 0.34, *p*-value = 0.80). Similarly, at 8-weeks neither the treatment effect (*F*(1,49) = 2.15, *p*-value = 0.15) or the treatment by time of day interaction were significant (*F*(3,49) = 0.60, *p*-value = 0.62). At 16-weeks neither the treatment effect (*F*(1,45) = 1.26, *p*-value = 0.27) or the treatment by time of day interaction were significant (*F*(3,45) = 1.56, *p*-value = 0.21).

For Females: at baseline neither the main effect for treatment (*F*(1,72) = 0.93, *p*-value = 0.34), or the treatment by time of day interaction were found to be significant (*F*(3,72) = 0.90, *p*-value = 0.44). Similarly, at 8-weeks neither the treatment effect (*F*(1,64) = 0.26, *p*-value = 0.61) or the treatment by time of day interaction were significant (*F*(3,64) = 0.82, *p*-value = 0.49). At 16-weeks neither the treatment effect (*F*(1,59) = 0.05, *p*-value = 0.82) or the treatment by time of day interaction were significant (*F*(3,59) = 0.81, *p*-value = 0.49).

#### 3.1.1. Waking Salivary Cortisol

In the sample as a whole, neither the main effect for treatment (*F*(1,131) = 0.02, *p*-value = 0.89), or the treatment by visit interaction (*F*(2,131) = 1.79, *p*-value = 0.17) were found to be significant. For males the main effect for treatment (*F*(1,55) = 0.83, *p*-value = 0.37), as well as the treatment by visit interaction (*F*(2,131) = 1.21, *p*-value = 0.31) were also non-significant. Similarly, for females neither the main effect for treatment (*F*(1,74) = 0.24, *p*-value = 0.63), or the treatment by visit interaction (*F*(2,74) = 2.02, *p*-value = 0.14) were found to be significant.

#### 3.1.2. Evening Salivary Cortisol

In the sample as a whole, neither the main effect for treatment (*F*(1,127) = 0.65, *p*-value = 0.42), or the treatment by visit interaction (*F*(2,127) = 2.17, *p*-value = 0.12) were found to be significant. For males the main effect for treatment (*F*(1,53) = 1.00, *p*-value = 0.32), as well as the treatment by visit interaction (*F*(2,53) = 0.88, *p*-value = 0.42) were also non-significant. Similarly, for Females neither the main effect for treatment (*F*(1,72) = 0.30, *p*-value = 0.58), or the treatment by visit interaction (*F*(2,72) = 0.88, *p*-value = 0.42) were found to be significant.

#### 3.1.3. Cortisol Awakening Response (CAR)

In the sample as a whole, the main effect for treatment (*F*(1,128) = 0.03, *p*-value = 0.86) was found to be non-significant, however the treatment by visit interaction was found to be significant (*F*(2,128) = 3.16, *p*-value = 0.045). Differences of Least Squares Means revealed that in the Placebo group from 8 to 16-weeks there was a near-significant *decrease* in the CAR value (*t*(128) = 1.8, *p*-value = 0.07, *d* = 0.22), while from baseline to 16-weeks there was a marginal *increase* in CAR (*t*(128) = −1.63, *p*-value = 0.11, *d* = 0.20) in the Multivitamin group. At 16-weeks the difference between CAR in the Multivitamin and Placebo groups was approaching significance (*t*(128) = −1.95, *p*-value = 0.054, *d* = 0.34).

For Males, the main effect for Treatment (*F*(1,53) = 0.34, *p*-value = 0.56) was found to be non-significant, however the treatment by visit interaction was significant (*F*(2,53) = 2.91, *p*-value = 0.047). Differences of Least Squares Means revealed that in the placebo group from Baseline to 16-weeks there was a trend towards a decrease in CAR (*t*(53) = 1.96, *p*-value = 0.055, *d* = 0.36), while CAR increased (non-significantly) over this same time period in the Multivitamin group (*t*(53) = −1.54, *p*-value = 0.13, *d* = 0.28).

**Figure 3:**
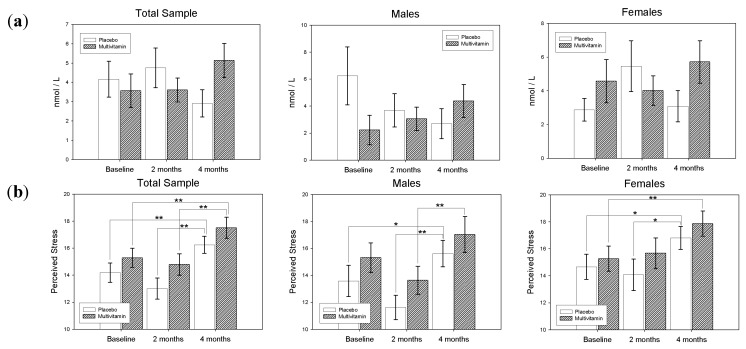
(**a**) Cortisol Awakening Response, and (**b**) Perceived Stress Scores (PSS) according to treatment group and gender at Baseline, 8-weeks and 16-weeks. Standard Error of the Means (SEM) displayed as error bars * *p* < 0.05, ** *p* < 0.01.

Similarly, for Females the main effect for Treatment (*F*(1,73) = 0.47, *p*-value = 0.49) was non-significant, and the treatment by visit interaction was also non-significant (*F*(2,73) = 1.30, *p*-value = 0.28). Differences of Least Squares Means revealed that CAR values remained relatively unchanged over the course of the study for both Multivitamin and Placebo groups. Differences in CAR values at baseline, 8-weeks and 16-weeks according to treatment group and gender are displayed in Figure 3.

### 3.2. Secondary Analysis: Relationships between Salivary Cortisol, Perceived Stress and Blood Levels of B Vitamins and Homocysteine

#### 3.2.1. Perceived Stress

Means (±SEMs) for scores on the PSS at baseline, 8-weeks and 16-weeks are displayed in Table 2 according to gender and treatment group. In the sample as a whole, both the main effect for treatment (*F*(1,135) = 2.03, *p*-value = 0.16) and the treatment by visit interaction (*F*(2,135) = 0.00, *p*-value = 0.99) was non-significant. However the main effect for visit was highly significant (*F*(2,135) = 15.88, *p*-value < 0.0001). Differences of Least Squares Means revealed a significant increase in PSS score from baseline to 16-weeks (*t*(135) = −3.15, *p*-value=0.002, *d* = 0.38) and from 8 to 16-weeks (*t*(135) = −3.89, *p*-value = 0.0002, *d* = 0.47) for the placebo group. Similarly, a significant increase in PSS was also observed from baseline to 16-weeks (*t*(135) = −3.19, *p*-value = 0.0018, *d* = 0.39) and from 8 to 16-weeks (*t*(135) = −3.87, *p*-value = 0.0002, *d* = 0.47) in the multivitamin group. However, no significant differences between groups were found at any time point.

For Males the main effect for treatment (*F*(1,57) = 0.99, *p*-value = 0.32) was also found to be non-significant, as well as the treatment by visit interaction (*F*(2,57) = 0.42, *p*-value = 0.66). However, the main effect for visit was found to be highly significant (*F*(2,57) = 18.9, *p*-value < 0.0001). Differences of Least Squares Means revealed a significant increase in PSS score from baseline to 16-weeks (*t*(57) = −2.57, *p*-value = 0.0128, *d* = 0.47) and from 8 to 16-weeks (*t*(57) = −4.19, *p*-value < 0.0001, *d* = 0.78) for the placebo group. For the multivitamin group, a significant decrease in PSS was observed from baseline to week 8 (*t*(57) = 2.54, *p*-value = 0.01, *d* = 0.46), while a significant increase in PSS was observed from 8 to 16-weeks (*t*(57) = −4.22, *p*-value < 0.0001, *d* = 0.77). However, no significant differences between groups were found at any time point.

Similarly, for Females the main effect for treatment (*F*(1,76) = 1.09, *p*-value = 0.30) was non-significant, and the treatment by visit interaction was also non-significant (*F*(2,76) = 0.35, *p*-value = 0.71). However, the main effect for visit was found to be significant (*F*(2,76) = 5.96, *p*-value < 0.0039). Differences of Least Squares Means revealed a significant increase in PSS score from baseline to 16-weeks (*t*(76) = −2.05, *p*-value = 0.01, *d* = 0.32) and from 8 to 16-weeks (*t*(76) = −2.19, *p*-value = 0.03, *d* = 0.35) for the placebo group. A significant increase in PSS was also observed from baseline to 16-weeks (*t*(76) = −2.68, *p*-value = 0.0089, *d* = 0.43) and a near-significant increase in PSS from 8 to 16-weeks (*t*(76) = −1.9, *p*-value = 0.06, *d* = 0.31) in the multivitamin group. However, no significant differences between groups were found at any time point. Differences in PSS scores according to treatment group and gender are displayed in Figure 3.

**Table 2:** Means and standard errors of Perceived Stress Scale (PSS) total and serum B6, B12, Red Cell Folate (RCF) and serum Homocysteine at Baseline, 8-weeks and 16-weeks according to treatment group.

Treatment			Baseline		8-Weeks		16-Weeks	
			*N*	M (SE)	*N*	M (SE)	*N*	M (SE)
*Perceived Stress Scale*								
	Total	Placebo	69	14.20 (0.72)	62	13.02 (0.78)	58	16.26 (0.64)
		MV	64	15.30 (0.70)	60	14.80 (0.79)	56	17.52 (0.78)
	Males	Placebo	30	13.60 (1.16)	27	11.63 (0.91)	27	15.63 (0.98)
		MV	28	15.32 (1.09)	26	13.65 (1.03)	24	17.04 (1.34)
	Females	Placebo	39	14.67 (0.93)	35	14.09 (1.17)	31	16.81 (0.85)
		MV	36	15.28 (0.94)	34	15.68 (1.14)	32	17.88 (0.94)
*Blood Measures*								
	*B6*	Placebo	50	114.08 (10.27)		121.32 (19.32)		130.84 (24.14)
		MV	43	111.70 (9.36)		631.40 (38.17)		567.67 (31.80)
	*B12*	Placebo	50	314.60 (14.48)		323.9000 (14.84)		318.9400 (15.46)
		MV	44	289.25 (12.11)		371.0909 (18.44)		391.9773 (15.32)
	*RCF*	Placebo	49	944.96 (21.94)		952.55 (26.09)		1039.65 (32.38)
		MV	41	933.27 (34.99)		1051.44 (46.11)		1237.05 (48.43)
	*HCy*	Placebo	51	10.6137 (0.22)		10.8725 (0.29)		10.4098 (0.33)
		MV	45	10.8444 (0.35)		9.8578 (0.27)		9.2244 (0.27)

Independent *t*-tests revealed that waking cortisol levels were not significantly different between high and low PSS groups (*t*(113) = −0.38, *p* = 0.70) at baseline. Similarly, evening cortisol levels were also not significantly different between high and low PSS groups (*t*(98) = 0.52, *p* = 0.60) at baseline, neither was CAR (*t*(93) = 1.01, *p* = 0.31).

#### 3.2.2. B-Vitamins and Homocysteine

Changes in B6, B12, RCF and HCy are displayed in Table 2 according to treatment group. Repeated measures ANCOVA adjusting for BMI revealed a significant interaction between study visit (baseline, 8-weeks, 16-weeks) and treatment group (multivitamin *versus* placebo) for B6 (pyridoxine) serum levels *F*(2,180) = 115.875, *p*-value < 0.001. B6 serum levels were found to be significantly higher for the multivitamin group in comparison to the placebo group at both 8-weeks (*t*(90) = 12.33, *p*-value < 0.001, *d* = 2.56) and 16-weeks (*t*(90) = 11.01, *p*-value < 0.001, *d* = 2.29). Repeated measures ANCOVA adjusting for BMI also revealed a significant interaction between study visit and treatment group for serum B12 (cobalamin) levels *F*(2,182) = 22.327, *p*-value < 0.001. Serum B12 levels were found to be significantly higher for the multivitamin group in comparison to the placebo group at both 8-weeks (*t*(91) = 2.00, *p*-value = 0.049, *d* = 0.41) and 16-weeks (*t*(91) = 3.34, *p*-value = 0.001, *d* = 0.69). Repeated measures ANCOVA adjusting for BMI revealed a significant interaction between study visit and treatment group for Red Cell Folate (RCF) levels *F*(2,174) = 9.503, *p*-value < 0.001. RCF levels were found to be significantly higher for the multivitamin group in comparison to the placebo group at 16-weeks (*t*(87) = 3.50, *p*-value = 0.001, *d* = 0.74). Repeated measures ANCOVA adjusting for BMI revealed a significant interaction between study visit and treatment group for serum homocysteine (HCy) levels *F*(2,186) = 8.852, *p* < 0.001. Serum HCy levels were found to be significantly lower for the multivitamin group in comparison to the placebo group at both 8-weeks (*t*(93) = 2.44, *p* = 0.015, *d* = 0.51) and 16-weeks (*t*(93) = 2.64, *p* = 0.009, *d* = 0.55).

Zero-order Pearson’s correlations between the B-vitamin and HCy blood measures, cortisol parameters and Perceived Stress at baseline are displayed in Table 3. No significant correlations between the B-vitamins and HCy blood measures, cortisol parameters and Perceived Stress scores were found. However, a trend level positive correlation between Baseline PSS and RCF level was observed (*r* = 0.148, *p* = 0.098). Partial correlations (controlling for baseline measures) between B-vitamins and HCy with salivary cortisol parameters and Perceived Stress at 8 and 16-weeks are displayed in Table 3. For B6, a trend level correlation with CAR was found at 16-weeks, after controlling for baseline B6 and CAR (*r* = 0.205, *p* = 0.074). For RCF, a significant correlation with CAR was found at 16-weeks, after controlling for baseline RCF and CAR (*r* = 0.268, *p* = 0.019). For B12, a trend level correlation with evening cortisol level was found at 16-weeks (*r* = −0.218, *p* = 0.060). For HCy, a significant correlation with waking cortisol was found at 8-weeks, after controlling for baseline HCy and waking cortisol (*r* = 0.313, *p* = 0.003).

**Table 3:** Correlations between B-vitamins and homocysteine with salivary cortisol parameters and Perceived Stress. Baseline values are zero-order correlations. 8-week and 16-week values are partial correlations (controlling for baseline measures). *p*-Values greater than 0.10 are displayed as non-significant (ns).

Study Visit		Waking		Evening		CAR		PSS	
		*r*	*p*	*r*	*p*	*r*	*p*	*r*	*p*
**B6**	*baseline*	0.015	ns	0.121	ns	0.032	ns	0.054	*ns*
	*8-weeks*	−0.002	ns	−0.100	ns	−0.092	ns	−0.051	*ns*
	*16-weeks*	−0.138	ns	−0.142	ns	**0.205** ^+^	**0.074**	0.050	*ns*
**RCF**	*baseline*	0.042	ns	0.059	ns	0.047	ns	**0.148** ^+^	**0.098**
	*8-weeks*	−0.177	ns	−0.095	ns	−0.097	ns	−0.057	*ns*
	*16-weeks*	−0.019	ns	−0.060	ns	**0.268** *	**0.019**	0.151	*ns*
**B12**	*baseline*	−0.031	ns	0.077	ns	−0.087	ns	0.130	*ns*
	*8-weeks*	0.013	ns	−0.085	ns	−0.102	ns	−0.125	*ns*
	*16-weeks*	−0.124	ns	**−0.218** ^+^	**0.060**	0.091	ns	−0.058	*ns*
**HCy**	*baseline*	0.022	ns	−0.132	ns	0.112	ns	−0.135	*ns*
	*8-weeks*	**0.313** **	**0.003**	−0.036	ns	−0.081	ns	0.052	ns
	*16-weeks*	−0.046	ns	−0.187	ns	0.035	ns	−0.045	ns
**PSS**	*baseline*	0.046	ns	−0.032	ns	−0.136	ns	-	-
	*8-weeks*	−0.061	ns	−0.081	ns	0.090	ns	-	-
	*16-weeks*	0.064	ns	0.144	ns	0.046	ns	-	-

^+^ *p* < 0.10; * *p* < 0.05; ** *p* < 0.01; RCF—Red cell folate; HCy—Homocysteine (serum); B6—Pyridoxine (serum); B12—Cyanocobalamin (serum); CAR—Cortisol Awakening Response.

## 4. Discussion

This study investigated the effects of chronic multivitamin supplementation on diurnal patterns of cortisol secretion and perceived stress. In contrast to previous studies of multivitamin supplementation and stress [15,16,20], 16-weeks supplementation with multivitamins was not found to be associated with a significant effect on self-report perceived stress. However, for reasons that are unclear a significant increase in perceived stress was reported across both treatment groups at 16-weeks in comparison to 8-weeks. In consideration of the fact that this change was seen equally in both groups, the evidence suggests that an external factor other than the multivitamin treatment was responsible for elevated stress levels over the last 8-weeks of the study. A possible reason for the lack of significant effect is that participants were in a state of low chronic stress at baseline and for this reason multivitamin treatment could only lower stress levels by a small amount. In the study by Lovell *et al*. [6], participants in the low stress group had a mean PSS score of 11.4, while participants in the high stress group had a mean PSS score of 21.1. This is in contrast to the current study where PSS mean values were in the range of 14 to 18 throughout the course of the study. Further, the selection of participants in the current study is in contrast to the study by Schlebusch *et al*. [15] where participants were specifically selected on the basis of high stress levels. It is also noteworthy that perceived stress levels were found to be unrelated to cortisol secretion parameters at baseline, which contrasts with the study by Lovell *et al*. [6]. This can also be interpreted as evidence to suggest that perhaps participants at baseline were range-limited to the lower end of the PSS dimension.

Notwithstanding the lack of treatment effect on perceived stress, the significant interaction between treatment group and study visit observed for CAR is intriguing. In this regard, the CAR was found to decrease in the placebo group from 8 to 16-weeks, while the CAR in the multivitamin group was found to increase over this same time period. While these changes were only at trend level (*p* < 0.10) it is interesting to note that exploratory analysis in the combined sample revealed that at 16-weeks the CAR was significantly positively correlated with RCF levels and also positively correlated with serum B6 levels at trend level (*p* < 0.10).

A number of cross-sectional studies have reported an association between an elevated CAR and a range of stress indicators including worry, work overload and self-reported stress [36,37,38,39]. In a systematic review and meta-analysis of CAR and its association with psychosocial factor, Chida and Steptoe [40] provided extensive evidence to suggest that an elevated CAR is often associated with job stress and general life stress (*r* = 0.061 and *r* = 0.065), while a blunted CAR is associated with fatigue, burnout and exhaustion (*r* = −0.065) or post-traumatic stress disorder (*r* = −0.141). An increased CAR is therefore, often observed in individuals at time of increased burden. However, more recently this increase has been interpreted as an adaptive response to everyday demands [41,42]. In support, Powell *et al*. [42] reported that higher CAR increases on waking were associated with subsequent attenuation of distress ratings in response to stressors experienced throughout the day, with the authors interpreting the increased CAR as being associated with successful coping. Further evidence has also been provided by the findings of greater CAR on weekdays, compared to weekends [43], and on the morning of an anticipated ballroom dancing competition compared to a training day [44]. Similarly, a recent longitudinal study by Izawa *et al*. [45] found that a prolonged two-week stressful situation was associated with initial CAR increase, followed by decreased CAR following the stressful period. In contrast, in the previous study by Lovell *et al*. [6] a significantly reduced CAR (*d* = 0.60) was found in participants with high levels of perceived stress, while in a recent study by Walker *et al*. [46], CAR AUC_G_ was found to be negatively associated with both anticipatory and trait anxiety.

Indices of the CAR are also related to processes that would allow an individual to prepare for forthcoming events, for example, a larger mean CAR was found to be associated with lower fatigue levels over a 3-day period in a study by Adam *et al*. [47] and levels of post-awakening arousal are positively associated with the CAR [48]. Research by Dahlgren *et al*. [49] also reported higher levels of sleepiness associated with lower levels of cortisol 15 min after awakening in healthy office workers. In consideration of the fact that salivary cortisol measurements for the current study were taken on the days preceding study visits, it could be assumed that these days were reasonably similar in terms of demands, *i.e.*, acute effects were not unduly influencing chronic changes. It is also important to note that a significant increase in perceived stress was reported for both the placebo and multivitamin group from 8 to 16 weeks. For these reasons, an elevation in CAR which emerged after PSS was reported to increase could be interpreted as evidence of greater adaptation to the everyday demands in those taking multivitamins.

Whilst the effect sizes of the changes in CAR that were found in the current study were only small (an increase in the multivitamin group of *d* = 0.20 from 8 to 16 weeks, a difference between treatment groups at week 16 of *d* = 0.34, and an increase in the male multivitamin group from baseline to 16-weeks of *d* = 0.28), these effects are comparable in magnitude to other between-group studies in the literature. For example, in clinical studies Powell *et al*. [50] reported a reduction in CAR of effect size *d* = 0.34 for patients with chronic fatigue syndrome in comparison to controls, while Manthey *et al*. [51] reported a reduction in the CAR of *d* = 0.34 for tricyclic antidepressant users in comparison to non-users, and Vreeburg *et al*. [52] reported an increase in the CAR of effect size *d* = 0.15 for remitted depression sufferers in comparison to controls. In non-clinical examples of more transient changes to the CAR, Doane *et al*. [53] conducted a study of jet lag, in which they found an increased CAR of magnitude *d* = 0.19 for participants who travelled eastward in comparison to those that did not cross any time zones, while Brand *et al*. [54] reported a reduction in CAR of magnitude *d* = 0.35 for participants after taking part in an 8-week meditation course. Similarly, when the Pearson’s *r* values from the meta-analysis by Chida and Steptoe [40] are converted to Cohen’s *d* values, values range from *d* = 0.12 for the effect of general life stress on the CAR, to *d* = −0.28 for the effect of post-traumatic stress disorder. At this stage, a direct comparison of the present findings to other nutritional intervention studies is not possible, due to the scarcity of research in this area. Further research regarding the effects of nutritional supplementation on the CAR is needed in order to establish reliable estimates of effects sizes associated with specific dietary supplements. 

In regards to a possible mechanism of action whereby multivitamin supplementation may influence the CAR response, the current finding of an association between increased B6 and folate levels and the CAR response at 16-weeks is a novel and intriguing result. In particular, the finding that changes in HCy were uncorrelated with CAR response at 16-weeks suggests that the mechanism of action was not attributable to HCy change. A possible mechanism by which B-vitamins could directly or indirectly effect HPA-axis regulation without HCy is via effects on methylation and monoamine metabolism [27], although further research is required to directly explore this possibility. While no significant associations between HCy and CAR were found, it is interesting to note that at 8-weeks, waking cortisol levels were positively correlated with HCy. A possible interpretation is that for those participants who still had elevated HCy levels at 8-weeks, waking levels of cortisol were also higher.

The interpretation of the current findings need be tempered by some important limitations. Firstly, while B-Vitamins were one of the main constituents included in the Swisse F1^®^ formula, there were a number of other constituents included in the supplement which could also foreseeably have impacted neuro-endocrine function, including anti-oxidants such as Vitamins C and E, and small quantities of selected botanicals. For this reason the effects on the CAR cannot be solely attributed to B-Vitamins. For example, whilst not investigated for its effects on the CAR directly, vitamin C has been found to attenuate cortisol secretion at a dosage of 1500 mg/day in long distance runners [55]. However, a much lower dosage of only 165 mg of vitamin C was present in the current multivitamin formulations for both males and females. The botanical *Ginkgo Biloba*, which was present in the male multivitamin formula at 100 mg, has also previously been found to ameliorate cortisol release in response to an acute stressor in healthy volunteers, using a single dose of 120 mg [56]. Similarly, *Eleutherococcus Senticossus* (*Siberian ginseng*) which was present in the female multivitamin formula in a small dose (25 mg) has a long history of use in Russia for the amelioration of stress under conditions of increased demands [57]. However, a recent study by Schaffler *et al*. [58] reported no additional change in the CAR following eight weeks of supplementation when a much larger daily dose of *Siberian ginseng* (120 mg/day) was administered in addition to stress management training. Korean/*Panax ginseng*, which was present in the male multivitamin formula (50 mg), also has evidence of ameliorating cortisol release in animal research [59], although these findings, together with appropriate dosages, are yet to be confirmed in human trials. While these findings suggest that possible effects of ingredients in addition to B-vitamins cannot be entirely ruled out, it is important to highlight that the levels of B-vitamins included in both the male and female multivitamin formulas were of high dosage in comparison to the other constituents. For example, the proportion of the recommended daily intakes (RDI) for the main B-vitamin constituents were as follows: vitamin B6; 53 times RDI (women)/49 times RDI (men), vitamin B12; 21 times RDI (women)/12.5 times RDI (men), and vitamin B9/folate; 1.25 times RDI (both men and women). These proportions are in contrast to levels of minerals such as calcium, iron and magnesium which did not exceed 0.5 times the RDI [30].

Notwithstanding this caveat, the finding of correlation between CAR and serum B-vitamin levels in the blood in the current study provides strong evidence to suggest that B-vitamins were at least a necessary constituent required to modulate the CAR response. This interpretation is also supported by previous behavioural research which has provided considerable evidence to suggest that high-dose B-vitamin supplementation may have anti-stress effects in humans [22]. However, in future studies supplementation with B-vitamins in isolation or an extended blood sample which tests for other constituents also present in the multivitamin would help to better delineate the causative ingredients. Another limitation of the current study was that in order to minimize participant burden and reduce attrition, the measurement of diurnal cortisol was restricted to only 4 time points on one day: waking, 15 min, 30 min and bedtime. Whilst these sampling points allow for the assessment of several HPA indices, a greater number of time points on two consecutive days, *i.e.*, waking, 15-min, 30-min, 45-min, 60-min, 1200 h (midday) and bedtime would have helped to more accurately ascertain diurnal cortisol profiles [60].

In future research, the selection of a sample that reported above-average levels of occupational stress at baseline as well as the measurement of additional information with a known relationship to HPA function may also be warranted. Whilst the participants in the current study were required to be engaged in at least part-time work or higher education, it is foreseeable that a stronger effect of multivitamin supplementation on both perceived stress and diurnal cortisol secretion would have emerged if they had been experiencing higher levels of stress at baseline. In future research it may also be informative to capture additional information that may impact on HPA function such as other measures of chronic stress as well as sleep quality. Indeed, a recent review in the area suggests that the cortisol awakening response is associated with a number of subjective and objective measures of sleep quality [61].

It is worth noting that in recently published findings from our group [30] few differences between the two groups were found in this sample in regards to chronic general health and mood outcomes (including GHQ-28, POMS, Chalder fatigue). However, it is noteworthy that for the male participants at week-16 there was a trend towards an increase in state-trait anxiety (STAI-S) in response to an acute stressor in the MV group [30]. Whilst it is difficult to interpret the relationship between anxiety experienced in relation to an acute stressor and diurnal cortisol secretion that was sampled the day before, this finding is intriguing considering that a trend-level increase in CAR was also observed for male participants in the current study from baseline to week-16. In future research, cortisol response in relationship to an acute stressor on the same day of testing may provide further insight into the hypothesis that multivitamin supplementation may ameliorate the effects of acute stress [11,17]. Finally, in future research the acute effects of multivitamin supplementation on diurnal cortisol secretion could be more clearly dissociated from chronic effects by instructing a subgroup of participants to refrain from taking their treatment on the day of saliva collection. In consideration of the fact that participants took multivitamins each morning, presumably within the first hour of waking, it is possible that acute effects may have had additional influence on cortisol secretion.

## 5. Conclusions

In conclusion, the current study provided preliminary evidence to suggest that 16-week supplementation with a multivitamin containing high-dose B-vitamins did not differentially affect self-report perceived stress in comparison to placebo. However, supplementation was found to be associated with changes to the cortisol awakening response at 16-weeks. In light of the fact that serum levels of red cell folate and B6 (but not HCy) were found to be associated with the CAR response at 16 weeks, this provides evidence to suggest that the elevation in CAR was mediated by the effects of folate and B6. Further research is required in order to further investigate these relationships.
